# Death of a 29-Year-Old Male from Undifferentiated Sepsis

**DOI:** 10.1155/2016/2478924

**Published:** 2016-03-30

**Authors:** Kathryn A. Trebuss, Samantha Buttemer, Jeffrey S. Wilkinson, Josie Xu, John P. Rossiter, Kieran M. Moore

**Affiliations:** ^1^Queen's University, Kingston, ON, Canada K7L 3N6; ^2^University of Toronto, Toronto, ON, Canada M5S 1A8; ^3^Department of Pathology and Molecular Medicine, Queen's University and Kingston General Hospital, Kingston, ON, Canada K7L 3N6; ^4^Department of Family Medicine and Emergency Medicine, Queen's University, Kingston, ON, Canada K7L 3N6

## Abstract

Tumour necrosis factor alpha inhibitors, such as infliximab, and other biologic agents are associated with increased risk of opportunistic infection, including tuberculosis. Tuberculosis infections associated with infliximab tend to present atypically and can be difficult to diagnose, as they are more likely to manifest as extrapulmonary or disseminated disease. The authors report a case involving a 29-year-old male patient who died following 16 days of treatment for undifferentiated sepsis and who was found on autopsy to have widespread disseminated tuberculosis. Prior to the onset of illness, the patient had received infliximab for the treatment of Crohn's disease. Following discussion of the case, the authors review the definition of adverse events, provide a root cause analysis of the cognitive errors and breakdowns in the health care system that contributed to the reported outcome, and identify opportunities to address these breakdowns and improve patient safety measures for future cases.

## 1. Case Presentation

A 29-year-old male presented to a rural Emergency Department (ED) in Ontario with a two-week history of decreased appetite, diarrhea, and worsening lethargy, malaise, and confusion. The patient's family denied any complaint of cough, shortness of breath, neurological symptoms, neck stiffness, or dysuria. The patient's past medical history was remarkable for schizophrenia and chronic diarrhea following ileocecal bowel resection for Crohn's disease. His medications included divalproex, clozapine, quetiapine, azathioprine, infliximab, eltroxin, loperamide, and ferrous fumarate. Tuberculin skin testing (TST) was performed prior to starting infliximab; however, it was completed while the patient was being actively treated with azathioprine and prednisone. The patient was of Caucasian descent, was born in Canada, and had not come into contact with any group at high risk of tuberculosis (TB) infection. He had never visited Aboriginal reserves in Canada and had not recently travelled outside of the country. Initial bloodwork revealed elevated liver function tests with a predominant hepatocellular injury (moderate elevation of AST and ALT, with only mild increases in bilirubin and ALP). Lactate was mildly elevated. He was treated with a bolus of normal saline and a single dose of ceftriaxone prior to transfer to the Internal Medicine service at a tertiary care centre.

**Figure 1:**
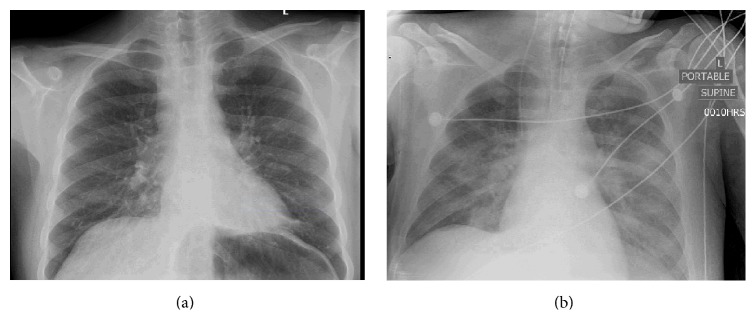


Upon arrival to tertiary care, the patient was alert but disoriented. His vital signs were as follows: blood pressure was 102/66 mmHg, heart rate was 85 beats/min, respiratory rate was 20 breaths/min, and oxygen saturation was normal on room air, but the oral temperature was quite elevated at 38.9°C. He had no rash, meningismus, or focal neurological findings. Blood work on admission showed a leukocyte count of 5.2 × 10^9^/L, mild anemia (Hb 117 × 10^9^/L), and thrombocytopenia (platelets 116 × 10^9^/L). The neutrophil count was within normal limits at 4.21 × 10^9^/L but the lymphocyte count was reduced at 0.52 × 10^9^/L; monocytes were also within normal limits at 0.31 × 10^9^/L. All electrolytes were normal. His previously elevated lactate levels had normalized to 1.7 mmol/L, but both the AST and ALT remained elevated at 323 U/L and 89 U/L, respectively. An HIV test was negative. The admission chest X-ray was reported to be suboptimal due to low lung volumes but was otherwise unremarkable (Figure 1(a)). The patient's ECG was normal, and a CT scan of the abdomen without contrast was negative for microperforations secondary to Crohn's exacerbation. Given the normal chest X-ray on admission, a CT scan of the chest was not completed.

The patient was admitted to the Medicine service with a diagnosis of sepsis and multiorgan dysfunction. He was treated with IV fluids for volume depletion and ceftriaxone for undifferentiated sepsis. On the second day of admission, the patient was transferred to the ICU due to a highly elevated temperature of 39.0°C with severe delirium and agitation. He was sedated, intubated, and treated with fluid resuscitation and vasopressors (norepinephrine) in accordance with the Adult Sepsis Management Pathway of the Surviving Sepsis Campaign [1].

In consultation with gastroenterology, azathioprine was discontinued due to lack of evidence for Crohn's exacerbation and concern that the patient may be septic due to immunocompromise. Psychiatrists were also consulted, and they recommended holding all antipsychotic medications to address the possibility that the patient's illness could be neuroleptic malignant syndrome (NMS), a rare but serious adverse effect of this class of drugs.

The patient's condition continued to deteriorate. Over the following days, the patient developed worsening hypotension requiring further vasopressor support, and his bloodwork showed pancytopenia, lactic acidosis, and multiorgan dysfunction. Liver enzymes and creatine kinase rose dramatically (AST 751 U/L, ALT 131 U/L, ALP 68, total bilirubin 17, CK >3000 U/L). The patient remained sedated and intubated and was treated with fluids, cooling to 36.0°C, vasopressors, broadened antibiotic therapy, and acyclovir to cover for possible viral meningoencephalitis from Herpes Simplex Virus (HSV). The course of antibiotics that was administered was as follows: ceftriaxone as monotherapy in the initial days of admission; ceftazidime, ampicillin, and vancomycin when the patient's condition worsened and he was transferred to the ICU; and finally a combination of vancomycin, meropenem, and acyclovir early in the course of the ICU admission, which was continued until the time of his death.

Multiple blood cultures over the entire hospital admission did not isolate any causative organism. Endotracheal secretions were tested twice over the course of the patient's admission to ICU and were nondiagnostic. The pathology report for the first sample noted that it contained few squamous epithelial cells, few lower respiratory cells, and few yeast cells; the second contained a significant number of epithelial cells, representing oropharyngeal contamination. Acid-fast staining was negative. Multiple chest X-rays completed in the ICU were significant for perihilar confluent airspace and ground glass opacities extending into the lower lobes bilaterally (Figure 1(b); taken on day 11 of hospital admission). The differential diagnosis included edema and/or infection, but no significant change in the radiographic appearance was noted during the patient's ICU stay. A CSF sample was obtained and did not show any abnormalities of cell count, glucose, or protein. Consultants in gastroenterology, hematology, infectious disease, neurology, psychiatry, and critical care were unable to explain the patient's fever, severe liver dysfunction, or clinical decline in spite of these multiple investigations.

On day 14, the patient developed jaundice and a new palpable purpuric rash and required inotropes. Following further deterioration, the patient's family decided to pursue palliative care. On day 16, the patient was extubated and died shortly thereafter. The death certificate indicated sepsis and multiorgan failure.

**Figure 2:**
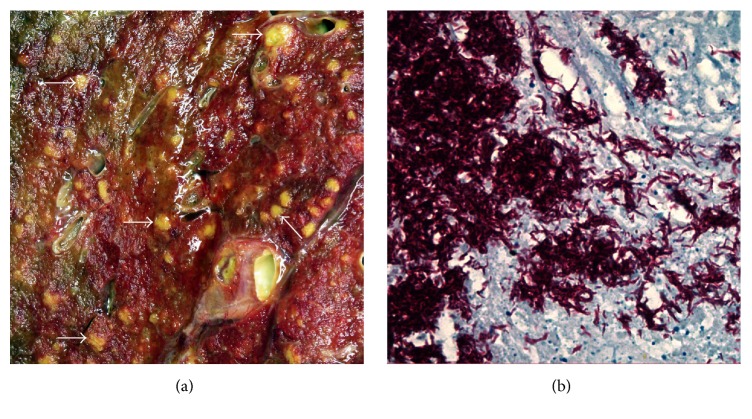
(a) Macroscopic image of an area (5.5 × 5.5 cm) of the cut surface of the left lung with numerous yellowish miliary (millet seed-like) lesions, representative examples of which are arrowed. (b) Photomicrograph of a necrotic area in a hilar lymph node containing numerous acid-fast bacilli (Ziehl-Neelsen stain; magnification ×450).

A consented autopsy was performed to elucidate a definitive diagnosis. The decedent was deeply icteric. Internal examination showed numerous miliary lesions in the lungs (Figure 2(a)), spleen, and liver and necrosis in enlarged hilar and paratracheal lymph nodes. Rapid tissue testing was positive for acid-fast bacilli, prompting a working diagnosis of disseminated tuberculosis infection. Hospital infection control and Public Health authorities were notified immediately. Polymerase Chain Reaction (PCR) testing confirmed the presence of* Mycobacterium tuberculosis*. Microscopy showed multifocal coagulative necrosis in the lungs (in association with extensive tuberculous pneumonia), thoracic lymph nodes, and other organs, especially spleen, liver, and bone marrow. These lesions contained acid-fast bacilli (Figure 2(b)) and infiltrates of neutrophils, lymphocytes, and numerous macrophages, but without granuloma formation. Macroscopic and microscopic examination of the bowel showed features of quiescent Crohn's disease but no evidence of TB. Public Health officials contacted the regional coroner and requested a coroner investigation, as this was considered an avoidable death.

## 2. Discussion

Infliximab (Remicade) is a chimeric (part human, part mouse) monoclonal antibody that inhibits tumour necrosis factor alpha (TNF-alpha), an important proinflammatory cytokine [2]. It is approved in Canada and the United States for treatment of inflammatory bowel disease and certain rheumatologic conditions and is administered by IV at 2- to 8-week intervals [3].

The risk of infection associated with infliximab and other anti-TNF-alpha agents is well documented. The frequency of tuberculosis, however, uniquely exceeds that of other serious opportunistic infections in patients treated with this particular TNF-alpha inhibitor [4, 5]. Although the precise mechanism by which infliximab increases susceptibility to tuberculosis is not yet fully understood, TNF-alpha is known to be involved in the recruitment of inflammatory cells, macrophage activation, granuloma formation, and disease containment. Inhibition of TNF-alpha may prevent compartmentalization of viable* M. tuberculosis* and orderly induction of macrophage apoptosis after bacillary ingestion and thereby facilitate widespread (re)activation and dissemination of latent or newly acquired disease [4, 6].

The manifestations of tuberculosis infection in patients on infliximab tend to be unusual, making diagnosis challenging. One study that reviewed data from the US Food and Drug Administration's Adverse Event Reporting System found that, of individuals on infliximab who were diagnosed with tuberculosis, 56 percent were found to have extrapulmonary disease and 24 percent had disseminated disease [4]. In contrast, among cases of tuberculosis not associated with HIV infection, approximately 18 percent manifest as extrapulmonary disease and disseminated disease accounts for less than 2 percent [4].

In order to diagnose tuberculosis, one must first suspect it, even without obvious risk factors or clear clinical signs. The appropriate investigations must also be ordered, including tissue analysis of the affected organs, since tuberculin skin tests, chest X-rays, and sputum samples may be falsely negative for patients who are immunosuppressed or have disseminated or extrapulmonary disease [7]. In the case above, bone marrow and liver biopsies likely would have been positive; however, these tests were never performed, because tuberculosis was not included on the differential diagnosis and the patient was at high risk of bleeding.

Due to its atypical presentation in those taking infliximab, physicians may easily fail to consider or adequately investigate a diagnosis of tuberculosis when seeing a patient with undifferentiated sepsis. Unless physicians are educated about the association between infliximab and tuberculosis and measures implemented to remind them of this association when they prescribe the medication or treat a patient taking it, patients treated with infliximab remain vulnerable to serious preventable adverse events.

Adverse events (AEs) are defined as “unintended injuries or complications resulting in death, disability, or prolonged hospital stay that arise from health care management” [8]. A major chart review published in the Canadian Medical Association Journal (CMAJ) in 2004 estimated that the AE rate per 100 hospital admissions in Canada was 7.5 and that as many as 36.9 percent of these may be highly preventable [8].

Diagnostic errors make up a significant proportion of documented AEs and commonly occur as a result of breakdown in both clinical reasoning and the overarching healthcare system [9]. Breakdowns at both levels contributed to the outcome of the case summarized above. The case was presented at the hospital's Morbidity and Mortality Rounds. The most significant errors that were identified there were as follows:

1. Many specialists were involved in the patient's care; however no case conference was ever convened to discuss the patient in a comprehensive manner. Each specialist was focused on their own field.
2. There was a failure to biopsy organs which were showing significant dysfunction with no clear mechanism of injury (e.g., no liver biopsy was ever completed).
3. The initial consultation by the infectious disease service failed to note that the patient had been previously treated with infliximab (since the medication was recorded by the electronic health record as stopped on admission, in spite of the fact that the medication continues to exert its effects between injections). The chart contained this information; however it was difficult to find, since it was only recorded on the initial medicine admission note. When the infectious disease team saw the patient, he had already been in hospital for more than a week, and his chart had grown significantly in size.
4. The TB skin test completed prior to initiating treatment with infliximab was done under nonideal circumstances, as the patient was undergoing active treatment with azathioprine and prednisone at the time of testing. There was no thought given to ordering an Interferon Gamma Release Assay (IGRA), a test which some consider to be a superior test for immunocompromised patients.

To prevent AEs from occurring in the future, it is essential to understand precisely why they occurred and what safeguards might prevent them. A root cause analysis (RCA) can help to establish the furthest upstream causes of an AE. Although many tools to facilitate RCA exist, for this case we chose to use the 5 Whys Tool, an iterative question-asking technique designed to elucidate root causes of a problem [10]. Using this tool, we identified several primary root causes that likely contributed to the outcome of the case above, all of which should be addressed to enhance patient safety (Table 1).

In this case, diagnostic error can be attributed to a number of systemic factors, as well as to challenges intrinsic to the clinical reasoning process. The human mind is inherently vulnerable to cognitive biases, logical fallacies, false assumptions, and other errors in thinking and reasoning, known collectively as cognitive failure [11]. Clinicians are by no means immune to such failures of thought. Experienced clinicians often use reflexive intuitive processes based on pattern recognition in diagnosis and management of patients, reserving the much slower analytical approach for more complex cases. Intuition is usually but not always correct, and these automatic processes are particularly prone to cognitive bias, even in the most seasoned clinicians [12]. While there is no time to assess each patient in an analytical manner, it is crucial that physicians remain vigilant for potential biases in their reasoning process and routinely reflect on the thought processes underlying their clinical decision making.

Groopman and Hartzband propose three simple questions to facilitate a more analytical approach to cases and enhance clinical reasoning (Table 2) [12]. By asking these questions routinely, clinicians can avoid a multitude of cognitive errors that contribute to diagnostic errors and AEs, including premature closure bias, anchoring bias, and diagnosis momentum.

## 3. Closure of Case

Following the death of the patient, Public Health identified a close contact with a 6-month history of chronic cough, who tested positive for pulmonary tuberculosis in spite of having no known risk factors. Public Health referred the case to the coroner for further investigation. Thereafter, the case was presented at the hospital's Morbidity and Mortality Rounds and referred to the Patient Safety Committee and the Infection Prevention and Control Committee.

The lessons of this case can be productively applied to all areas of clinical practice. This case serves as a sobering reminder of the significant clinical consequences that can result from cognitive bias and other forms of cognitive failure. It functions as well to illuminate the utility of RCA in assessing AEs and modifying those elements of care that may have contributed to their occurrence.

Biological agents that act by suppressing components of the immune system are becoming an increasingly important part of routine care, and it is imperative that all physicians are aware of the risks associated with these potent medications, in particular the risk of presenting atypically with certain life-threatening infections. Sustained postmarketing surveillance is needed to monitor the AEs associated with these (and other) drugs. It behooves us all to be active participants in this process to protect our patients. MedEffect Canada, the Health Canada reporting system for drug safety, tracks AEs submitted voluntarily, but more work is needed to ensure thorough data collection [13].

Hospitals, too, have a responsibility to improve safety for patients taking immunosuppressants, which are becoming an increasingly common treatment modality in a growing number of medical specialties. We recommend that hospitals identify by bracelet and in the chart all patients taking drugs that cause immunosuppression at the time of admission and that they develop a system for flagging specific infection risks associated with particular immunosuppressants. We recommend as well that immunosuppressants which patients are receiving at regular intervals but not daily or while in hospital be included on the medications list, as these medications continue to exert their effects on patients between doses and must therefore be considered throughout the clinical decision making process. More generally, we recommend that hospitals take steps to integrate all components of patients' medical records, so that critical information is not lost during transfers, and facilitate regular interdisciplinary conferences for clinicians involved in shared cases.

Finally, in the event of a death from undifferentiated sepsis, it is essential to perform an autopsy to determine where possible the mechanism of disease responsible. Clinicians should discuss the case with Public Health if there is concern that a death may have been caused by a reportable disease and/or with the coroner's office if there is concern about the medical care provided, in order to determine whether or not to mandate an autopsy if the decedent's family is reluctant or unwilling to consent to the procedure.

Clinicians can advocate for such changes within their hospital and adopt a more analytical approach to cases that incorporates Groopman and Hartzband's three questions. Those prescribing MABs and other immune-suppressing medications should also consider prescribing medical alert bracelets with these drugs, especially those administered by IV over long intervals, as patients may neglect to include these on medication lists when they present to secondary and acute care.

## Figures and Tables

**Table 1:** RCA for death of 29-year-old male from disseminated tuberculosis.

Problem	Patient died of undiagnosed disseminated tuberculosis				
Why?	Tuberculosis not on differential diagnosis				

Why?	Cognitive bias during diagnosis		Lack of awareness of infliximab use and associated adverse effects		

Why?	No interspecialty case conference	No tool used to prevent bias during diagnosis	No centralized medication listing across hospitals	No red-flagging for high-risk medications	Incomplete handover between physicians during transfers

Why?				Incomplete medication postmarketing surveillance	

**Table 2:** Forms of cognitive failure addressed by Groopman and Hartzband's questions.

What else could this be?	Is there something that does not fit?	Is there more than one diagnosis?
Reminds physician to think widely and consider rare but significant diagnoses; can prevent *premature closure bias*, that is, settling on a diagnosis before it has been adequately substantiated by clinical evidence	Reminds physician to consider whether each data point fits with proposed diagnosis; can prevent *anchoring bias*, that is, the tendency not to reevaluate a diagnosis even when presented with information that contradicts it	Reminds physician that conditions can coexist; can prevent anchoring bias and *diagnosis momentum*, that is, trend for clinicians not to challenge a diagnosis once established
